# Determinants of Insecticide-Treated Net Utilization for Malaria Prevention Among Under-Five Children in The Gambia: Evidence From National Survey

**DOI:** 10.1155/jotm/6340482

**Published:** 2025-04-14

**Authors:** Amadou Barrow, Bakary Kinteh, Mansour Badjie, Amadou Kongira, Ayodeji Matthew Adebayo, Rex A. Kuye, Edrisa Sanyang

**Affiliations:** ^1^Department of Epidemiology, University of Florida, Gainesville, Florida, USA; ^2^Department of Public and Environmental Health, School of Medicine and Allied Health Sciences, University of The Gambia, Kanifing, Gambia; ^3^School of Public Health, Gambia College, Brikama Campus, Brikama, West Coast Region, Gambia; ^4^Department of Community Medicine, University of Ibadan, Ibadan, Nigeria; ^5^Center for Environmental and Workplace Health, Department of Public Health, College of Health and Human Services, Western Kentucky University, Bowling Green, Kentucky, USA

**Keywords:** determinant, insecticide-treated net, The Gambia, under-five children

## Abstract

**Background:** Malaria is one of the deadliest mosquito-borne diseases. Despite the demonstrated benefits of insecticide-treated nets (ITNs) usage in children under 5 years of age, nonuse is linked to higher mortality and morbidity rates. This study examined how child-, maternal-, household-, and community-level determinants influence ITN utilization among children under 5 in The Gambia for malaria prevention. It further elucidates how household environment, infrastructure, and drinking water sources mediate under-5 ITN utilization in The Gambia.

**Method:** Secondary data analysis of The Gambia Demographic Health Survey (2019–2020) was conducted in this study. We used Chi-square test, linear model ANOVA, multivariable regression model, and mediation analysis to analyze the influence of child-, maternal-, household-, and community-level factors on under-5 ITN utilization in The Gambia. We computed crude and adjusted odds ratios (cOR and aOR, respectively) for potential confounders across groups, with statistical significance set at *p* < 0.05, and 95% confidence interval (CI).

**Results:** The prevalence of ITN utilization among under-5 children was 63.4% (95% CI: 61.0%, 65.8%). This study identified several significant factors influencing under-5 ITN utilization in The Gambia, such as children's anemia status (aOR = 0.53, 95% CI [0.27, 0.97], *p* = 0.050), mother's literacy (aOR = 0.77, 95% CI [0.61, 0.96], *p* = 0.021), female household heads (aOR = 0.67, 95% CI [0.56, 0.81], *p* < 0.001), household wealth index (aOR = 0.55, 95% CI [0.42, 0.72], *p* < 0.001), and residence (aOR = 1.30, 95% CI [1.04, 1.62], *p* = 0.022). Ethnicity and region also influenced ITN utilization, with variations across different ethnic groups and regions (including Kerewan aOR = 2.29, 95% CI [1.54, 3.39], *p* < 0.001). Mediation analysis highlighted both the direct and indirect effects of household infrastructure and drinking water sources on ITN utilization, emphasizing the multifaceted nature of the factors influencing ITN use in this context.

**Conclusion:** This study elucidates the complex factors influencing ITN utilization among children under 5 years of age in The Gambia. The nuanced understanding of individual-, household-, and community-level factors offers a robust foundation for targeted strategies for malaria prevention, with far-reaching implications for public health policy and practice.

## 1. Introduction

Insecticide-treated nets (ITNs) have been recognized as an effective tool in the global fight against malaria, a disease that continues to plague many parts of the world, particularly Africa. According to World Health Organization's latest World Malaria Report, there were an estimated 263 million malaria cases and 597,000 deaths worldwide by 2023, representing an increase of approximately 11 million cases compared to 2022, while death rates remain relatively stable [1]. The WHO African Region continues to bear the heaviest burden, accounting for approximately 95% of global deaths, with many at-risk populations still lacking access to essential prevention, detection, and treatment services [1, 2]. Despite these challenges, WHO data have revealed that an estimated 2.2 billion cases of malaria and 12.7 million deaths have been averted since 2000, although the disease remains a serious global health threat [1]. Although the utilization of ITNs has significantly increased in recent years, disparities in access and usage remain a challenge. In Africa, approximately 56% of households possess at least one ITN in 2021, with usage rates remaining suboptimal at 41.2% for children under 5 and 50% for pregnant women, highlighting the persistent need for enhanced ITN coverage and utilization strategies [3–5].

The global strategy to combat malaria aligns with the Sustainable Development Goals (SDGs), particularly Goal 3, which seeks to ensure healthy lives and promote the well-being of people of all ages. Various interventions have been targeted for malaria control in Africa, with ITN distribution being the cornerstone. For instance, Roll Back Malaria (RBM) Partnership has emphasized the need for universal access to ITNs to achieve a significant reduction in malaria incidence and mortality by 2030 [6]. These international efforts underscore the importance of ITN utilization in achieving broader health and development objectives.

In The Gambia, children under -5 are identified as particularly vulnerable to malaria [7]. A recent national survey revealed significant regional variations in ITN utilization among under-5 children, with usage highest in Janjanbureh (60%) and lowest in Basse (34%) [8]. It is noteworthy that ITN use in this vulnerable age group has remained stagnant, with no significant changes since 2013 [8, 9], despite massive decades of behavior-change campaigns to promote utilization. The battle against malaria in The Gambia is influenced by various factors and determinants that influence ITN utilization. According to national statistics, the prevalence of malaria among children under 5 years of age was only 0.2% in 2019 [8]. Previous studies have pinpointed key factors affecting ITN use, including household income, education level, and awareness of malaria prevention measures [10–13]. Furthermore, the National Malaria Control Strategy and related health policies have played an instrumental role in molding the landscape of ITN utilization in The Gambia [14]. The significant reduction of prevalence rates at regional and national levels could be attributed to these policies and malaria interventions such as indoor residual spraying (IRS), intermittent preventive treatment for pregnant women (IPTp), seasonal malaria chemoprevention (SMC), ITNs, and case management [14].

The rationale for this study lies in the need to further understand specific determinants of ITN utilization among children under 5 in The Gambia. Although previous research has provided valuable insights, there remains a gap in the comprehensive understanding of complex interplay of factors influencing ITN uptake. By focusing on the unique context of The Gambia, this research could help advance the knowledge of ITN utilization, thereby supporting the region's broader goal of malaria prevention and control. Therefore, this study is being conducted to explore and analyze determinants of ITN utilization for malaria prevention among under-5 children in The Gambia, providing evidence that may guide future strategies and interventions.

## 2. Methods

### 2.1. Study Design, Data Source, and Study Population

This study was a secondary data analysis of the Gambia Demographic and Health Survey (GDHS) conducted between November 2019 and March 2020 [8]. The GDHS is a comprehensive and nationally representative health survey conducted every 5 years, employing a layered sampling strategy, standardized tools, and proficient interviewers. The DHS Program implemented rigorous quality control measures throughout the data collection and processing phases to ensure data accuracy and reliability. These include extensive interviewer training and standardization, coupled with a robust double-data entry system to minimize errors. During fieldwork, quality assurance was maintained through daily generation of field check tables to identify inconsistencies, real-time data quality monitoring using computer-assisted personal interviewing (CAPI), and systematic field supervision with spot checking of completed interviews. The survey design incorporated skip patterns and validation rules into the questionnaire, followed by thorough post-fieldwork data processing and editing procedures to ensure data consistency and completeness. The GDHS 2019–20 achieved a household response rate of 95%, with minimal missing data (< 5%) for the variables included in this analysis, and data quality assessments conducted by DHS Program indicated good internal consistency and reliability of the survey data [8]. The principal sample unit of the survey was formed from randomly chosen cluster samples.

For this study, the 2019/20 GDHS Children Recode (KR) data file was used, focusing on the assessment of ITN utilization among children under the age of five. A total of 25 households were systematically selected from 143 clusters across 8 Local Government Areas (LGAs). The response rate was 95% [8]. The sample included 3810 under-5 children across all regions. The data were curated using GDHS 2019/20 dataset.

### 2.2. Variable Selection and Measurement

#### 2.2.1. Outcome Variable

The dependent variable was under-5 children who slept under an ITN before the survey. In this study, ITNs included both long-lasting insecticidal nets (LLINs) that were pretreated by manufacturers and conventional ITNs that required periodic re-treatment, as categorized in GDHS 2019–20 survey. The binary indicator was set to 1 if a child slept under an ITN (either LLIN or conventional ITN) and 0 otherwise [15, 16].

#### 2.2.2. Covariates/Explanatory Variables

Covariates/explanatory variables were categorized into child-, mother-, household-, and community-level factors.

At child level, we considered the child's anemic status (severe, moderate, mild, or not anemic) and number of under-5 children in the household. Maternal-level variables included marital status (not in union, in union, or married), place of delivery (home or institutional health facility), current pregnancy status (yes or no), maternal age in years, literacy (cannot read at all or can at least read or write), educational level (no formal education, primary education, or secondary/higher education), and current working status (yes or no).

Household-level variables included age and sex of household head, household wealth index, drinking water sources, and main materials used for the floor, walls, and roof. The main floor, wall, and roof materials were also dichotomized into improved and unimproved categories according to WHO classification [17, 18]. The selection of these variables was guided by both theoretical frameworks of health behavior and previous literature on environmental determinants of malaria prevention practices [19–21]. Drinking water sources were included, as they serve as a proxy for household infrastructure and overall health awareness, potentially influencing preventive health behaviors, including ITN use. Additionally, water sources can indicate areas of standing water that may increase the local malaria transmission risk, making households more or less likely to prioritize ITN use. These sources were dichotomized into improved (safe) and unimproved (unsafe) based on available recommendations and guidelines [15, 16, 18]. Similarly, housing materials were included as they reflect both socioeconomic status and physical barriers to mosquito entry, which may influence the perceived need for ITN use.

Community-level variables included residence (urban or rural), region (Banjul, Basse, Brikama, Janjanbureh, Kanifing, Kerewan, Kuntaur, or Mansakonko), cluster altitude in meters, and ethnicity (Mandinka/Jahanka, Wollof/Serere, Fula/Tukulur/Lorobo, Jola/Sarahule, or non-Gambian/Others).

### 2.3. Statistical Analysis

The analysis was performed using SAS version 9.4 (SAS Institute, NC, Cary, USA.) and R version 4.2.1 including its survey package (v4.0) to account for the complex sampling design of DHS data. The following steps were performed for each analysis: (i) examining the risk factors for under-5 children's utilization of ITNs at the child, maternal, household, and community levels, and (ii) conducting mediation analysis to examine the potential mediating effects of household infrastructure and drinking water sources on the utilization of ITNs among under-5 children in The Gambia.

First, bivariate analyses were performed to explore the associations between dependent and independent variables using the Pearson Chi-square test of independence for categorical variables and linear model ANOVA for continuous variables (Table 1). Variables incorporated into the multivariate model (Table 2) were analyzed to determine their independent effects while controlling for other factors. The selection of variables for mediation analysis was based on the Akaike information criterion (AIC), utilizing the STEPAIC function of the MASS R package (v7.3–53.1) [22]. The analysis involved assessing the indirect and total effects of risk factors on ITN utilization through the proposed mediators (household infrastructure and drinking water sources). Collinearity diagnostic tests were employed to assess multicollinearity among the explanatory variables using tolerance levels (< 0.1) and variance inflation factors (≤ 10). We developed several regression models to analyze the associations between ITN utilization and potential determinants: (1) a child-level factor model, (2) a maternal-level factor model, (3) a household-level factor model, and (4) a community-level factor model. Finally, we constructed a full multivariable logistic regression model incorporating all significant variables from previous models. For each model, we calculated adjusted odds ratios (aOR) and their corresponding 95% confidence intervals (CIs) to indicate the direction and strength of the associations, as well as mediation effects. Statistical significance was set at *p* < 0.05.

### 2.4. Ethical Considerations

The DHS received ethical clearance from the Medical Research Council/Gambia Government Joint Research Ethics Committee as indicated by the reference number/ID SCC1570v1.1. Furthermore, permission from the DHS Program to utilize the Gambia DHS dataset (https://www.dhsprogram.com/data/available-datasets.cfm) for our research was secured. Following the protocol of informed consent upheld by the DHS Program, we adhered to the principles of anonymity and confidentiality to ensure proper use of the data.

## 3. Results

### 3.1. Individual, Demographic, and Contextual Characteristics

The analysis revealed several significant patterns across child-, maternal-, household-, and community-level characteristics. As shown in Table 1, at the child level, anemia status was significantly associated with ITN utilization (*p* < 0.001), with severely anemic children having the highest ITN usage (80.6%) compared to those with moderate (67.5%), mild (61.1%), or no anemia (62.1%). Maternal characteristics showed notable variations in the use of ITN. Literacy status was significantly associated with ITN utilization (*p* < 0.001), with higher usage among mothers who could not read or write (65.6%) compared to literate mothers (57.5%). Similarly, maternal education showed significant differences (*p* < 0.001), with higher ITN utilization among mothers with no formal education (66.5%) compared to those with primary (60.3%) or secondary/higher education (59.9%).

Household-level factors were strongly associated with ITN use. The sex of the household head was significantly related to ITN utilization (*p* < 0.001), with male-headed households showing higher usage (65.0%) compared to female-headed households (54.0%). A clear socioeconomic gradient was observed in the household wealth index (*p* < 0.001), with ITN usage decreasing as wealth increased: poorest (71.6%), poorer (67.8%), middle (60.3%), richer (55.3%), and richest (43.8%). Housing characteristics also showed significant associations with higher ITN usage in households with unimproved walls (71.5% vs. 60.9% for improved, *p* < 0.001), unimproved floors (67.7% vs. 62.5% for improved, *p*=0.014), and unimproved roofs (70.4% vs. 62.3% for improved, *p* < 0.001).

Community-level factors demonstrate strong geographical and cultural variations. Rural residents showed significantly higher ITN utilization (70.5%) compared to urban residents (54.8%, *p* < 0.001). Regional differences were substantial (*p* < 0.001), with Kerewan showing the highest ITN usage (77.1%) and Kanifing showing the lowest (48.9%). Ethnic variations were also significant (*p* < 0.001), with the Mandinka/Jahanka group showing higher utilization rates (66.2%) compared to other ethnic groups, particularly the Jola/Karoninka group, which showed the lowest usage (50.0%).

### 3.2. Association Between Under-5 ITN Utilization and Child-Level Factors

In the regression analysis predicting under-5 ITN utilization, child-level factors such as anemia status and the number of children under-5 in the household were found to be significant predictors (Table 2). After controlling for other factors in the multivariate analysis, children with severe anemia had the highest likelihood of ITN use, serving as the reference category. Compared with children with severe anemia, those with moderate anemia were 44% less likely to use ITNs (aOR = 0.56, 95% CI [0.29, 0.99]), those with mild anemia were 56% less likely (aOR = 0.44, 95% CI [0.23, 0.78]), and those without anemia were 54% less likely (aOR = 0.46, 95% CI [0.25, 0.82]). These results suggest that ITN utilization was the highest among children with severe anemia, possibly reflecting an increased awareness of malaria prevention needs in households with severely anemic children. The number of children under 5 years of age in a household was inversely related to ITN utilization, with each additional child decreasing the likelihood of ITN use (aOR = 0.97, 95% CI [0.95, 1.00]). These findings underscore the importance of considering anemia status and household composition in strategies aimed at improving ITN utilization among under-5 children.

### 3.3. Association Between Under-5 ITN Utilization and Maternal-Level Factors

In predicting under-5 ITN utilization, several maternal-level factors were found to be significant in predicting under-5 ITN utilization. Several inverse relationships were observed in the regression analysis. Mothers who could read and write were 23% less likely to use ITNs compared to those who could not (aOR = 0.77, 95% CI [0.67, 0.90]). Similarly, children of mothers with primary education showed 13% lower odds of ITN use compared to those whose mothers had no education (aOR = 0.87, 95% CI [0.77, 0.99]). Households with female heads demonstrated 17% lower odds of ITN utilization compared to male-headed households (aOR = 0.83, 95% CI [0.72, 0.95]). These findings highlight important sociodemographic disparities in ITN utilization that warrant further investigation.

### 3.4. Under-5 ITN Utilization Association With Household-Level Factors

In the multivariable regression analysis examining household-level factors influencing under-5 ITN utilization, several noteworthy relationships were observed. Households with female heads were more likely to use ITNs than those with male heads (aOR = 0.83, 95% CI [0.72, 0.95]). However, the age of the household head did not significantly affect ITN utilization (aOR = 1.00, 95% CI [0.99, 1.00]). The household wealth index was a significant factor. Compared to households in the middle wealth index, the richest households were less likely to utilize ITNs (aOR = 0.59, 95% CI [0.49, 0.70]), as were richer households (aOR = 0.66, 95% CI [0.57, 0.77]). By contrast, the poorest households were more likely to use ITNs (aOR = 1.37, 95% CI [1.19, 1.58]). Households with unimproved wall materials were more likely to use ITNs than those with improved materials (aOR = 1.21, 95% CI [1.04, 1.41]). However, neither the quality of the main floor materials nor the roof materials significantly influenced ITN utilization (aOR = 1.08, 95% CI [0.91, 1.28] and aOR = 1.00, 95% CI [0.82, 1.21], respectively). Lastly, households with unimproved drinking water sources were less likely to use ITNs compared to those with improved sources (aOR = 0.75, 95% CI [0.63, 0.88]).

### 3.5. Under-5 ITN Utilization Association With Community-Level Factors

In a multivariable regression analysis examining community-level factors influencing under-5 ITN utilization, several significant relationships emerged that align with known malaria transmission patterns in The Gambia. Compared to rural residences, households in urban areas were more likely to utilize ITNs (aOR = 1.59, 95% CI [1.39, 1.81]), even though urban areas generally have lower transmission intensity due to reduced vector breeding sites. Regional analysis using Banjul as a reference revealed varying patterns of ITN utilization that corresponded to local ecological and transmission characteristics. Kerewan, characterized by its riverine ecology and high transmission intensity (annual EIR > 20), showed significantly higher odds of ITN use (aOR = 1.83, 95% CI [1.39, 2.40]). Similarly, Kuntaur, with its extensive rice irrigation schemes creating vector breeding sites, demonstrated higher utilization (aOR = 1.68, 95% CI [1.27, 2.23]). Janjanbureh, featuring mixed savannah and wetland ecosystems that support year-round transmission, showed the highest odds of ITN use (aOR = 2.10, 95% CI [1.57, 2.79]). Mansakonko also showed higher utilization (aOR = 1.34, 95% CI [1.00, 1.77]), while Basse, despite its rural setting, showed no significant difference (aOR = 0.78, 95% CI [0.60, 1.02]).

Environmental factors played a clear role, with an inverse relationship observed between cluster altitude and ITN utilization (aOR = 0.97, 95% CI [0.97, 0.98]). This pattern reflects vector ecology, as lower-lying areas typically have higher humidity and more breeding sites, supporting a greater mosquito density. Ethnicity also significantly influenced ITN utilization, possibly reflecting different cultural practices and settling patterns across ecological zones. Both Wollof/Sarahule (aOR = 0.85, 95% CI [0.76, 0.95]) and Jola (aOR = 0.73, 95% CI [0.60, 0.90]) ethnic groups were less likely to use ITNs than Mandinka. These findings indicate that ITN utilization patterns are shaped by a complex interplay of geographical, environmental, and cultural factors that align with the known malaria transmission dynamics in The Gambia.

### 3.6. Predictors of Under-5 ITN Utilization in The Gambia

**Figure 1:**
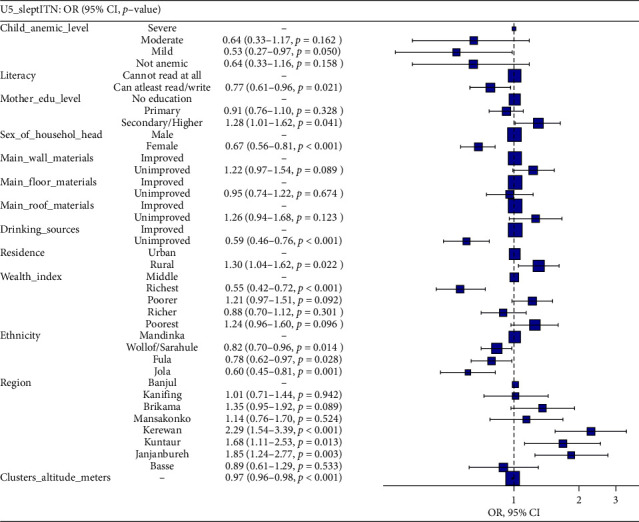
Predictors of under-5 ITN utilization in the Gambia 2019/20. *Note:* This figure presents variables that remained statistically significant in the final multivariable model after applying the Akaike information criterion (AIC) for model selection. Some variables that appear in Table 2 preliminary models (such as child BMI and number of under-5 children in the household) were excluded from this visualization, as they did not meet the statistical threshold for inclusion in the final model based on the STEPAIC function results. Model fit statistics: AIC = 5115.3, C-statistic = 0.68.

In a full multivariable regression model (Figure 1) predicting under-5 ITN utilization, numerous factors were significantly associated with the outcome. The severity of anemia in children was inversely associated with ITN utilization, with mild anemia (aOR = 0.53, 95% CI [0.27, 0.97], *p*=0.050) presenting a significant association. Literacy had a positive effect on ITN utilization, with those who could read or write more likely to use ITNs (aOR = 0.77, 95% CI [0.61, 0.96], *p*=0.021). Mothers with secondary or higher education levels were more likely to use ITNs (aOR = 1.28, 95% CI [1.01, 1.62], *p*=0.041). Households headed by females were more likely to utilize ITNs (aOR = 0.67, 95% CI [0.56, 0.81], *p* < 0.001). Households with unimproved drinking water sources were less likely to use ITNs (aOR = 0.59, 95% CI [0.46, 0.76], *p* < 0.001). Compared with urban residence, rural residence was associated with higher ITN utilization (aOR = 1.30, 95% CI [1.04, 1.62], *p*=0.022).

The richest households were less likely to use ITNs than were middle-income households (aOR = 0.55, 95% CI [0.42, 0.72], *p* < 0.001). Ethnicity also significantly influenced ITN utilization, with Wollof/Serer (aOR = 0.82, 95% CI [0.70, 0.96], *p*=0.014), Fula (aOR = 0.78, 95% CI [0.62, 0.97], *p*=0.028), and Jola (aOR = 0.60, 95% CI [0.45, 0.81], *p*=0.001) ethnic groups being less likely to use ITNs than Mandinka. In terms of region, Kerewan (aOR = 2.29, 95% CI [1.54, 3.39], *p* < 0.001), Kuntaur (aOR = 1.68, 95% CI [1.11, 2.53], *p*=0.013), and Janjanbureh (aOR = 1.85, 95% CI [1.24, 2.77], *p*=0.003) were significantly more likely to utilize ITNs. As altitude increased, the likelihood of ITN use decreased slightly (aOR = 0.97, 95% CI [0.96, 0.98], *p* < 0.001). This model was adequately fitted, as indicated by an AIC of 5115.3 and a C-statistic of 0.68.

### 3.7. Mediation Analysis of Indirect and Total Effects of Household Infrastructures, Drinking Water Sources on Under-5 ITN Utilization

**Figure 2:**
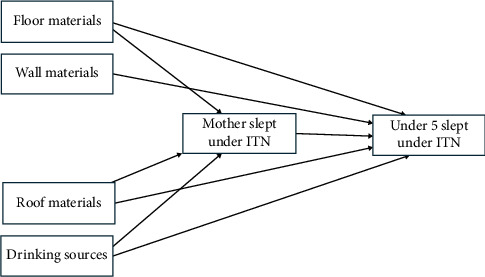
Conceptual path diagram.

The results of the mediation analysis (as shown in Table 3 and graphically presented in Figure 2) employing the standard delta method demonstrated both the direct and indirect effects of different household variables on the utilization of ITNs by children under the age of 5. There were significant indirect effects of whether the mother slept under a bed net on the main floor materials (*β* = 0.03613, SE = 0.0136, *p* < 0.001), main wall materials (*β* = 0.05475, SE = 0.0119, *p* < 0.001), and drinking water sources (*β* = −0.03625, SE = 0.0140, *p* < 0.001). In contrast, the main roof materials had a significant negative indirect effect (*β* = −0.02926, SE = 0.0155, *p*=0.007).

Direct effects were found for the main wall materials (*β* = 0.02399, SE = 0.0108, *p*=0.013) and main roof materials (*β* = 0.03227, SE = 0.0141, *p*=0.001), whereas no significant direct effect was found for the main floor materials (*β* = −4.89e − 4, SE = 0.0123, *p*=0.960) and drinking water sources (*β* = −0.00545, SE = 0.0127, *p*=0.501).

The total effects revealed significant associations for main floor materials (*β* = 0.03564, SE = 0.0184, *p* = 0.014), main wall materials (*β* = 0.07874, SE = 0.0160, *p* < 0.001), and drinking water sources (*β* = −0.04170, SE = 0.0189, *p* < 0.001). However, the main roof material had no significant total effect (*β* = 0.00301, SE = 0.0209, *p* = 0.837). These results highlight the importance of considering both direct and indirect pathways through which different aspects of the household environment may impact the utilization of ITNs in children under 5.

## 4. Discussion

This study found that 63.4% (95% CI: 61.0%, 65.8%) of children under 5 in The Gambia slept under an ITN, falling short of the World Health Organization's universal coverage target of 80%. This prevalence is higher than that in many other West African countries, such as Nigeria, where 41.2% of children under 5 slept under an ITN, with regional disparities influenced by socioeconomic factors [4]. Similarly, ITN utilization in Ghana was reported to be 54%, whereas Senegal achieved a higher prevalence of 71% [23]. The observed utilization rate in The Gambia represents both progress and persistent challenges in malaria prevention efforts. While this rate has improved from historical levels, it indicates that approximately one-third of vulnerable children remain unprotected from malaria transmission during sleep. This gap in coverage is particularly concerning given The Gambia's continued inclusion in high-burden malaria countries despite recent reductions in prevalence. The utilization rate varies substantially across regions, from as high as 77.1% in Kerewan to as low as 48.9% in Kanifing, highlighting significant geographical disparities in ITN use that require targeted interventions [19, 24].

The determinants of utilization span child and maternal attributes to household characteristics and community structures. In The Gambia, ITN utilization for children under 5 years of age shows marked variation, with challenges including availability of treated nets, affordability, and culturally rooted beliefs that may impede utilization rates [7, 14, 24]. Some of the significant factors in the analysis were child-anemic levels, mothers' literacy and education, sex of the household heads, and the household wealth index. All these factors were significantly associated with ITN use. These findings are consistent with those of previous studies conducted in other African countries such as Nigeria, Ghana, Liberia, and Ethiopia, where anemia has been identified as a key determinant of ITN utilization [7, 25–27]. For instance, both Awantang et al. and Abate et al. found that anemia severity was a motivating factor for the likelihood of ITN utilization [26, 28].

Maternal-level factors such as literacy and education were significant predictors of ITN utilization in The Gambia. This finding is consistent with those of studies from Liberia, where maternal education significantly influenced health-related behaviors, including malaria prevention strategies [27]. Our findings revealed an unexpected negative association between maternal literacy and ITN utilization, with literate mothers being 23% less likely to use ITNs compared to those who could not read or write. This finding contrasts with much of the existing literature from other African contexts, where literacy is often positively associated with preventive health behaviors [4] The lower ITN utilization among literate mothers in The Gambia might reflect complex socio-cultural factors, such as different risk perceptions or use of alternative malaria prevention methods in more educated households [28]. It may also suggest that current ITN promotion programs have been particularly effective in reaching and convincing nonliterate mothers about the importance of ITN use, possibly through targeted community-based interventions and pictorial health education materials [5]. This finding challenges assumptions about the straightforward relationship between literacy and health behaviors, suggesting the need for more nuanced approaches to ITN promotion that consider the diverse needs and perspectives of both literate and nonliterate populations.

Our study found a significant inverse relationship between household wealth and ITN utilization in The Gambia, with the poorest households showing 37% higher odds of ITN use compared to middle-wealth households, while the richest households were 41% less likely to use ITNs. This pattern contrasts with findings from Ethiopia, where wealthier households exhibit higher ITN usage rates [29], highlighting the context-specific nature of socioeconomic influences on ITN utilization. The higher utilization among poorer households in The Gambia might reflect the success of targeted distribution programs and behavior-change interventions in reaching economically disadvantaged populations. These findings underscore the multifaceted influences of maternal education, wealth, and living conditions on ITN utilization, advocating tailored intervention strategies that address these diverse determinants. By considering the unique socioeconomic and cultural contexts of each region, malaria prevention programs can more effectively target the underlying factors that influence ITN use, thereby maximizing their impact on reducing malaria incidence.

Our findings revealed significant associations between household leadership and ITN utilization, with children in female-headed households being 17% less likely to use ITNs compared to those in male-headed households. This finding contrasts with some studies from other African contexts, where female household leadership is often positively correlated with child health interventions [24]. The lower ITN utilization in female-headed households in The Gambia might reflect broader socioeconomic vulnerabilities, as these households often face greater resource constraints and may have limited access to health information networks [4]. This finding suggests the need for targeted support and empowerment programs for female-headed households to enhance their capacity to implement malaria-prevention measures.

The study also identified a significant relationship between drinking water sources and ITN utilization, with households using unimproved water sources being 25% less likely to use ITNs. This association likely reflects the broader patterns of health awareness and access to basic infrastructure [19]. Households with access to improved water sources may have better overall health awareness and greater access to public health services, including malaria prevention programs [5]. The mediation analysis further revealed that drinking water sources had significant indirect effects (*β* = −0.03625, SE = 0.0140, *p* < 0.001) on ITN utilization, suggesting that water source quality serves as an indicator of broader socio-environmental conditions that influence health behaviors. This finding emphasizes the interconnected nature of public health infrastructure and malaria prevention practices, suggesting that improvements in basic infrastructure might have positive spillover effects on ITN utilization [24].

Community-level factors, including residence type, regional location, altitude, and ethnicity, also significantly affected ITN utilization. The pronounced impact of ethnicity on ITN usage underscores the pivotal role that cultural beliefs, practices, and social norms play in shaping health behaviors, as echoed in comparative studies across the African continent [7]. These cultural dimensions can either facilitate or hinder malaria prevention efforts, suggesting that successful intervention strategies must be culturally sensitive and context specific. Moreover, mediation analysis not only delineates the pathways through which household characteristics influence ITN usage but also underscores the complexity of these relationships [14]. By revealing both direct and indirect effects, it highlights how structural factors, such as the physical attributes of homes, intertwine with socioeconomic and cultural elements to shape ITN utilization patterns. This complexity necessitates a multifaceted approach to policy formulation and intervention design that integrates structural improvements with targeted educational campaigns and community engagement initiatives to address the nuanced determinants of ITN use [14]. Such an integrated approach promises to enhance the efficacy of malaria prevention strategies, catering to the diverse needs and contexts of affected populations and paving the way for more sustainable health outcomes.

The geographical patterns of ITN utilization in The Gambia reveal complex spatial disparities that warrant careful consideration. Our findings showed significantly higher ITN utilization in rural areas (70.5%) compared to urban areas (54.8%), with rural residents having 1.30 times higher odds of ITN use (95% CI [1.04, 1.62]). This rural–urban disparity may reflect several factors. Rural areas in The Gambia typically experience higher malaria transmission rates due to environmental conditions that are more conducive to mosquito breeding, which may increase the perceived risk and motivation for ITN use [24]. Rural areas have been the primary focus of many ITN distribution campaigns and malaria prevention programs, potentially leading to better coverage and awareness in these regions [4]. Regional variations were particularly striking, with ITN utilization ranging from 77.1% in Kerewan to 48.9% in Kanifing. Notably, regions such as Kerewan, Kuntaur, and Janjanbureh showed significantly higher odds of ITN use compared to the reference region. These regional disparities likely reflect a combination of factors, including variations in malaria transmission intensity, differences in healthcare infrastructure, the effectiveness of local health promotion programs, and socio-cultural factors [5]. The lower utilization rates in more urbanized regions, such as Kanifing, may be partially explained by lower perceived malaria risk in urban settings, despite evidence suggesting significant urban malaria transmission in The Gambia [23]. Moreover, the inverse relationship between cluster altitude and ITN utilization suggests that environmental factors play a crucial role in shaping ITN use patterns, with communities at lower elevations showing higher utilization rates, possibly due to greater mosquito abundance in these areas [19].

Furthermore, the findings also underscore the significant indirect effects of maternal ITN use on the choice of main floor and wall materials and drinking water sources. This highlights the nuanced interplay between maternal behavior and environmental factors related to ITN utilization. Our results are consistent with those from Nigeria, Myanmar, and Ethiopia [13, 16, 29]. Conversely, direct effects suggest that the material of the main walls and roof directly influences ITN use among children, indicating direct impact of the structural components of the household on malaria prevention efforts. The absence of significant direct effects for the main floor materials and drinking water sources emphasizes the complexity of factors influencing ITN use. The total effects analysis further delineated the significant associations between household characteristics and ITN utilization, affirming the critical role of both direct and indirect pathways in enhancing malaria prevention strategies for vulnerable populations [30]. These findings highlight the multifaceted influences on ITN utilization in children under 5, underscoring the importance of integrated approaches in improving malaria prevention in The Gambia.

In contrast, a study conducted in Kenya found that health education had a significant positive effect on ITN utilization, highlighting the effectiveness of education and awareness campaigns in improving malaria prevention practices [31]. While socioeconomic factors such as income and parental education were associated with higher ITN use, health education emerged as a strong independent predictor [32]. These findings contrast with those from The Gambia, where household infrastructure and maternal behavior played a more significant role, suggesting that while structural and environmental factors are crucial, the impact of education and awareness should not be underestimated. This finding underscores the need for integrated malaria prevention strategies that combine structural improvements with targeted health education, particularly in rural settings.

Our findings on housing structures and ITN utilization, particularly that households with unimproved walls were more likely to use ITNs, warrant further investigation from an entomological perspective. While our study establishes these associations, understanding the underlying mechanisms requires detailed entomological studies examining how different housing materials affect mosquito entry, resting behavior, and ultimately, malaria transmission risk. Similar integrated studies conducted in other African contexts have provided valuable insights. In Tanzania, Kirby et al. [33] demonstrated that improved housing with screened windows and closed eaves reduced indoor mosquito density by 87% compared with traditional houses. In Mozambique, Kampango et al. [34] found that house structure modifications, particularly roof design and eave closure, significantly affect both mosquito entry patterns and ITN effectiveness. A systematic review by Tusting et al. [35] across sub-Saharan Africa showed that improved housing could reduce malaria infection and transmission by up to 47%, highlighting the importance of considering both housing improvements and ITN use in malaria control strategies. Future research combining epidemiological and entomological approaches in The Gambia could investigate (1) how different wall, roof, and floor materials influence indoor mosquito density and species composition; (2) the interaction between housing improvements and ITN effectiveness; and (3) the relative contribution of housing structure versus ITN use to malaria prevention in different ecological settings. Such integrated studies, similar to those conducted in Uganda by Wanzirah et al. [36] and in Malawi by McCann et al. [37], would provide valuable insights for optimizing both housing improvements and ITN interventions in The Gambia's specific context.

### 4.1. Strengths and Limitations

This study's utilization of Gambia's DHS dataset for secondary analysis is significant. These data are nationally representative and provide a comprehensive and robust source of information that enhances the generalizability of the findings. The extensive range of variables included in the DHS allowed for a multifaceted examination of the complex factors that influence ITN utilization. The use of the established statistical methods further ensured reliability of the results. The study's focus on both child and maternal attributes, household and community characteristics, and the inclusion of mediation analysis provided a nuanced understanding of the barriers and facilitators of ITN use in The Gambia.

Despite its strengths, this study had some limitations. The reliance on secondary data from DHS restricted the analysis to the variables and questions included in the original survey, potentially omitting other relevant factors that were not captured. The cross-sectional nature of DHS data means that causal relationships between variables cannot be definitively established. Although acknowledged as important, cultural beliefs and practices may not have been fully explored because of the limitations of the dataset. The use of self-reported data in the DHS may have introduced response bias, as participants might have reported socially desirable behaviors related to ITN usage. Finally, the study's focus on The Gambia may limit the applicability of the findings to other contexts with different malaria prevalence rates, healthcare systems, or socioeconomic conditions.

## 5. Conclusion

This study sheds light on the intricate factors that influence the utilization of ITNs among children under 5 years of age in The Gambia. These factors include child and maternal attributes and household and community characteristics, and underscore both the barriers and facilitators of ITN use. Despite ongoing efforts to expand ITN coverage, challenges, such as availability, affordability, and cultural beliefs, persist. A comprehensive approach is needed to enhance ITN utilization, encompassing continuous distribution, education on the benefits and proper usage of ITNs, and community engagement to address cultural barriers. Strengthening health systems and supply chains is vital to ensure consistent access to ITNs for children, thus improving their health outcomes and advancing the broader goal of malaria prevention in The Gambia. The findings of this study emphasize the need for targeted interventions that combine these strategies, as they are essential in boosting ITN utilization and contributing to the reduction of the malaria burden in the region.

## Figures and Tables

**Table 1:** Child-, maternal-, household-, and community-level characteristics of under-5 ITN utilization, DHS 2019/20 (*N* = 3810).

Variables	Child sleep under insecticide-treated nets (ITNs)			
	No (*N* = 1395)	Yes (*N* = 2415)	Total (*N* = 3810)	*p* value
Child-level factors				
Child anemic level				< 0.001^2^
Severe	12.0 (19.4%)	50.0 (80.6%)	62.0 (100%)	
Moderate	291.0 (32.5%)	604.0 (67.5%)	895.0 (100%)	
Mild	398.0 (38.9%)	626.0 (61.1%)	1024.0 (100%)	
Not anemic	694.0 (37.9%)	1135.0 (62.1%)	1829.0 (100%)	
Number under 5 in the household				0.092^1^
Mean (SD)	3.9 (3.1)	3.8 (2.6)	3.8 (2.8)	
Range	0.0–14.0	0.0–15.0	0.0–15.0	
Mother-level factors				
Marital status				0.083^2^
Not in union	80.0 (42.6%)	108.0 (57.4%)	188.0 (100%)	
In union/Married	1315.0 (36.3%)	2307.0 (63.7%)	3622.0 (100%)	
Place of delivery				0.536^2^
Home	276.0 (37.6%)	458.0 (62.4%)	734.0 (100%)	
Institutional	1119.0 (36.4%)	1957.0 (63.6%)	3076.0 (100%)	
Current pregnancy				0.189^2^
No or unsure	1251.0 (36.3%)	2197.0 (63.7%)	3448.0 (100%)	
Yes	144.0 (39.8%)	218.0 (60.2)	362.0 (100%)	
Age of mother				0.120^1^
Mean (SD)	29.5 (6.8)	29.9 (6.8)	29.7 (6.8)	
Range	15.0–49.0	15.0–49.0	15.0–49.0	
Literacy				< 0.001^2^
Cannot read/write at all	954.0 (34.4%)	1819.0 (65.6%)	2773.0 (100%)	
Can at least read/write	441.0 (42.5%)	596.0 (57.5%)	1037.0 (100%)	
Mother educational level				< 0.001^2^
No education	661.0 (33.5%)	1311.0 (66.5%)	1972.0 (100%)	
Primary	283.0 (39.7%)	429.0 (60.3%)	712.0 (100%)	
Secondary/Higher	451.0 (40.1%)	675.0 (59.9%)	1126.0 (100%)	
Mother currently working				0.743^2^
No	650.0 (36.9%)	1112.0 (63.1%)	1762.0 (100%)	
Yes	745.0 (36.4%)	1303.0 (63.6%)	2048.0 (100%)	
Household-level factors				
Sex of household head				< 0.001^2^
Male	1140.0 (35.0%)	2116.0 (65.0%)	3256.0 (100%)	
Female	255.0 (46.0%)	299.0 (54.0%)	554.0 (100%)	
Age of household head (in years)				0.064^1^
Mean (SD)	52.3 (15.4)	51.4 (15.1)	51.7 (15.2)	
Range	18.0–98.0	18.0–95.0	18.0–98.0	
Household wealth index				< 0.001^2^
Poorest	365.0 (28.4%)	922.0 (71.6%)	1287.0 (100%)	
Poorer	280.0 (32.2%)	590.0 (67.8%)	870.0 (100%)	
Middle	294.0 (39.7%)	447.0 (60.3%)	741.0 (100%)	
Richer	220.0 (44.7%)	272.0 (55.3%)	492.0 (100%)	
Richest	236.0 (56.2%)	184.0 (43.8%)	420.0 (100%)	
Main floor materials				0.014^2^
Improved	1187.0 (37.5%)	1980.0 (62.5%)	3167.0 (100%)	
Unimproved	208.0 (32.3%)	435.0 (67.7%)	643.0 (100%)	
Main wall materials				< 0.001^2^
Improved	1136.0 (39.1%)	1766.0 (60.9%)	2902.0 (100%)	
Unimproved	259.0 (28.5%)	649.0 (71.5%)	908.0 (100%)	
Main roof materials				< 0.001^2^
Improved	1248.0 (37.7%)	2065.0 (62.3%)	3313.0 (100%)	
Unimproved	147.0 (29.6%)	350.0 (70.4%)	497.0 (100%)	
Drinking water sources				0.059^2^
Improved	1241.0 (36.1%)	2194.0 (63.9%)	3435.0 (100%)	
Unimproved	154.0 (41.1%)	221.0 (58.9%)	375.0 (100%)	
Community-level factors				
Residence				< 0.001^2^
Urban	779.0 (45.2%)	945.0 (54.8%)	1724.0 (100%)	
Rural	616.0 (29.5%)	1470.0 (70.5%)	2086.0 (100%)	
Region				< 0.001^2^
Banjul	80.0 (41.2%)	114.0 (58.8%)	194.0 (100%)	
Kanifing	186.0 (51.1%)	178.0 (48.9%)	364.0 (100%)	
Brikama	292.0 (45.7%)	347.0 (54.3%)	639.0 (100%)	
Mansakonko	128.0 (34.4%)	244.0 (65.6%)	372.0 (100%)	
Kerewan	112.0 (22.9%)	378.0 (77.1%)	490.0 (100%)	
Kuntaur	114.0 (23.1%)	379.0 (76.9%)	493.0 (100%)	
Janjanbureh	120.0 (24.7%)	365.0 (75.3%)	485.0 (100%)	
Basse	363.0 (47.0%)	410.0 (53.0%)	773.0 (100%)	
Clusters altitude meters				< 0.001^1^
Mean (SD)	21.7 (10.2)	20.1 (10.4)	20.7 (10.3)	
Range	1.0–55.0	1.0–55.0	1.0–55.0	
Ethnicity				< 0.001^2^
Mandinka/Jahanka/Bambara	626.0 (33.8%)	1228.0 (66.2%)	1854.0 (100%)	
Wollof/Serer/Sarahule	503.0 (38.2%)	815.0 (61.8%)	1318.0 (100%)	
Fula/Tukulur/Lorobo	154.0 (37.2%)	260.0 (62.8%)	414.0 (100%)	
Jola/Karoninka/Creaole/Aku	112.0 (50.0%)	112.0 (50.0%)	224.0 (100%)	

^1^Linear Model ANOVA.

^2^Pearson's Chi-squared test.

**Table 2:** Association between under-5 ITN utilization, child-, maternal-, household-, and community-level factors.

Variables	Model I		Model II		Model III		Model IV	
	(Univariable) cOR (95% CI)	(Multivariable) aOR (95% CI)	(Univariable) cOR (95% CI)	(Multivariable) aOR (95% CI)	(Univariable) cOR (95% CI)	(Multivariable) aOR (95% CI)	(Univariable) cOR (95% CI)	(Multivariable) aOR (95% CI)
Child-level factors								
Child anemic status								
Severe	1	1						
Moderate	0.54 (0.28–0.96)^∗^	0.56 (0.29–0.99)^∗^						
Mild	0.42 (0.22–0.74)^∗^	0.44 (0.23–0.78)^∗^						
Not anemic	0.44 (0.23–0.77)^∗^	0.46 (0.25–0.82)^∗^						
Child BMI	1.01 (1.00–1.03)	1.00 (1.00–1.02)						
Number of U5 in household	0.96 (0.95–0.98)^∗^	0.97 (0.95–1.00)^∗^						
Maternal-level factors								
Marital status								
Not in union			1	1				
In union/Married			1.21 (0.99–1.48)	1.14 (0.92–1.39)				
Literacy								
Cannot read at all			1	1				
Can at least read/write			0.78 (0.70–0.86)^∗^	0.77 (0.67–0.90)^∗^				
Mother's educational level								
No education			1	1				
Primary			0.84 (0.74–0.95)^∗^	0.87 (0.77–0.99)^∗^				
Secondary/Higher			0.82 (0.74–0.91)^∗^	1.00 (0.86–1.17)				
Household-level factors								
Sex of household head								
Male					1	1		
Female					0.72 (0.63–0.82)^∗^	0.83 (0.72–0.95)^∗^		
Age of household head					0.99 (0.99–1.00)^∗^	1.00 (0.99–1.00)^∗^		
Household wealth index								
Middle					1	1		
Richest					0.56 (0.47–0.67)^∗^	0.59 (0.49–0.70)^∗^		
Poorer					1.12 (0.98–1.30)	1.12 (0.97–1.29)		
Richer					0.65 (0.56–0.76)^∗^	0.66 (0.57–0.77)^∗^		
Poorest					1.50 (1.32–1.71)^∗^	1.37 (1.19–1.58)^∗^		
Main floor materials								
Improved					1	1		
Unimproved					1.48 (1.30–1.67^∗^)	1.08 (0.91–1.28)		
Main wall materials								
Improved					1	1		
Unimproved					1.55 (1.39–1.73)^∗^	1.21 (1.04–1.41)^∗^		
Main roof materials								
Improved					1	1		
Unimproved					1.41 (1.22–1.62)^∗^	1.00 (0.82–1.21)		
Drinking water sources								
Improved					1	1		
Unimproved					0.98 (0.85–1.14)	0.75 (0.63–0.88)^∗^		
Community-level factors								
Residence								
Rural							1	1
Urban							1.83 (1.67–2.01)^∗^	1.59 (1.39–1.81)^∗^
Region								
Banjul							1	1
Kanifing							0.65 (0.51–0.83)^∗^	0.90 (0.70–1.17)
Brikama							0.64 (0.51–0.80)^∗^	1.01 (0.79–1.29)
Mansakonko							1.27 (0.98–1.64)	1.34 (1.00–1.77)^∗^
Kerewan							1.53 (1.20–1.95)^∗^	1.83 (1.39–2.40)^∗^
Kuntaur							1.69 (1.32–2.15)^∗^	1.68 (1.27–2.23)^∗^
Janjanbureh							1.77 (1.38–2.28)^∗^	2.10 (1.57–2.79)^∗^
Basse							0.57 (0.46–0.72)^∗^	0.78 (0.60–1.02)
Clusters altitude in meters							0.98 (0.98–0.98)^∗^	0.97 (0.97–0.98)^∗^
Ethnicity								
Mandinka							1	1
Wollof/Sarahule							0.77 (0.70–0.85)^∗^	0.85 (0.76–0.95)^∗^
Fula							0.83 (0.72–0.96)^∗^	0.88 (0.76–1.03)
Jola							0.53 (0.44–0.65)^∗^	0.73 (0.60–0.90)^∗^

*Note:* Model I: Child-level factors adjusted for child's anemic level, BMI and number of U5 in household; Model II: Maternal-level factors adjusted for marital status, literacy level, and maternal education; Model III: Household-level factors adjusted for sex & age of household head, wealth index, main floor, wall & roof materials, and drinking water source; Model IV: Community-level factors adjusted for residence, region, cluster's altitude, and ethnicity.

Abbreviations: aOR, adjusted odds ratio; cOR, crude odds ratio.

^∗^Statistical significance *p* < 0.05.

**Table 3:** Mediation analysis of indirect and total effects of household infrastructures, drinking water source, and under-5 ITN utilization.

Type	Effect	Estimate	SE	95% C.I. (a)		β	z	*p*
				Lower	Upper			
Indirect	Main floor materials1 ⇒ mother slept under bed net ⇒ under-5_sleptITN	0.046	0.014	0.019	0.072	0.036	3.358	< 0.001
	Main wall materials1 ⇒ mother slept under bed net ⇒ under-5_sleptITN	0.061	0.012	0.038	0.085	0.055	5.143	< 0.001
	Main roof materials1 ⇒ mother slept under bed net ⇒ under-5_sleptITN	−0.042	0.016	−0.072	−0.012	−0.029	−2.707	0.007
	Drinking water sources1 ⇒ mother slept under bed net ⇒ under-5_sleptITN	−0.057	0.014	−0.084	−0.029	−0.036	−4.062	< 0.001

Direct	Main floor materials1 ⇒ under-5_sleptITN	−6.18e − 4	0.012	−0.025	0.024	−4.89e − 4	−0.050	0.96
	Main wall materials1 ⇒ under-5_sleptITN	0.027	0.011	0.006	0.048	0.024	2.481	0.013
	Main roof materials1 ⇒ under-5_sleptITN	0.046	0.014	0.019	0.074	0.032	3.287	0.001
	Drinking_water_sources1 ⇒ under-5_sleptITN	−0.009	0.013	−0.033	0.016	−0.005	−0.672	0.501

Total	Main_floor_materials1 ⇒ under-5_sleptITN	0.045	0.018	0.009	0.081	0.036	2.454	0.014
	Main_wall_materials1 ⇒ under-5_sleptITN	0.088	0.016	0.057	0.119	0.079	5.483	< 0.001
	Main_roof_materials1 ⇒ under-5_sleptITN	0.004	0.021	−0.037	0.045	0.003	0.206	0.837
	Drinking_water_sources1 ⇒ under-5_sleptITN	−0.065	0.019	−0.102	−0.028	−0.042	−3.462	< 0.001

*Note:* Confidence intervals computed with method: Standard (Delta method). Betas are completely standardized effect sizes.

## Data Availability

The data that support the findings of this study are available in Demographic and Health surveys (DHS) at https://www.dhsprogram.com/data/available-datasets.cfm, reference number GAM-DHS-19/20. These data were derived from the following resources available in the public domain: MEASURE DHS, https://www.dhsprogram.com/data/available-datasets.cfm.
